# Can Tai Chi training impact fractal stride time dynamics, an index of gait health, in older adults? Cross-sectional and randomized trial studies

**DOI:** 10.1371/journal.pone.0186212

**Published:** 2017-10-11

**Authors:** Brian J. Gow, Jeffrey M. Hausdorff, Brad Manor, Lewis A. Lipsitz, Eric A. Macklin, Paolo Bonato, Vera Novak, Chung-Kang Peng, Andrew C. Ahn, Peter M. Wayne

**Affiliations:** 1 Osher Center for Integrative Medicine, Brigham and Women’s Hospital, Harvard Medical School, Boston, Massachusetts, United States of America; 2 Division of Interdisciplinary Medicine and Biotechnology, Beth Israel Deaconess Medical Center, Harvard Medical School, Boston, Massachusetts, United States of America; 3 Center for the Study of Movement, Cognition, and Mobility, Neurological Institute, Tel Aviv Sourasky Medical Center, Sagol School of Neuroscience and Sackler Faculty of Medicine, Tel Aviv University, Tel Aviv, Israel; 4 Division of Gerontology, Beth Israel Deaconess Medical Center, Harvard Medical School, Boston, Massachusetts, United States of America; 5 Institute for Aging Research, Hebrew SeniorLife, Roslindale, Massachusetts, United States of America; 6 Biostatistics Center, Massachusetts General Hospital, Harvard Medical School, Boston, Massachusetts, United States of America; 7 Motion Analysis Laboratory, Spaulding Rehabilitation Hospital, and the Department of Physical Medicine and Rehabilitation, Harvard Medical School, Boston, Massachusetts, United States of America; 8 Harvard-MIT Division of Health Sciences and Technology, Cambridge, Massachusetts, United States of America; 9 Department of Neurology, Beth Israel Deaconess Medical Center, Harvard Medical School, Boston, Massachusetts, United States of America; 10 Martinos Center for Biomedical Imaging, Massachusetts General Hospital, Charlestown, Massachusetts, United States of America; Midwestern University, UNITED STATES

## Abstract

**Purpose:**

To determine if Tai Chi (TC) has an impact on long-range correlations and fractal-like scaling in gait stride time dynamics, previously shown to be associated with aging, neurodegenerative disease, and fall risk.

**Methods:**

Using Detrended Fluctuation Analysis (DFA), this study evaluated the impact of TC mind-body exercise training on stride time dynamics assessed during 10 minute bouts of overground walking. A hybrid study design investigated long-term effects of TC via a cross-sectional comparison of 27 TC experts (24.5 ± 11.8 yrs experience) and 60 age- and gender matched TC-naïve older adults (50–70 yrs). Shorter-term effects of TC were assessed by randomly allocating TC-naïve participants to either 6 months of TC training or to a waitlist control. The alpha (α) long-range scaling coefficient derived from DFA and gait speed were evaluated as outcomes.

**Results:**

Cross-sectional comparisons using confounder adjusted linear models suggest that TC experts exhibited significantly greater long-range scaling of gait stride time dynamics compared with TC-naïve adults. Longitudinal random-slopes with shared baseline models accounting for multiple confounders suggest that the effects of shorter-term TC training on gait dynamics were not statistically significant, but trended in the same direction as longer-term effects although effect sizes were very small. In contrast, gait speed was unaffected in both cross-sectional and longitudinal comparisons.

**Conclusion:**

These preliminary findings suggest that fractal-like measures of gait health may be sufficiently precise to capture the positive effects of exercise in the form of Tai Chi, thus warranting further investigation. These results motivate larger and longer-duration trials, in both healthy and health-challenged populations, to further evaluate the potential of Tai Chi to restore age-related declines in gait dynamics.

**Trial registration:**

The randomized trial component of this study was registered at ClinicalTrials.gov (NCT01340365).

## Introduction

Gait is a fundamental, yet complex activity of daily living that requires the functional integration of skeletal, muscular, nervous, circulatory, and respiratory systems. Multiple characteristics of gait performance decline with age in older adults, and this decline is associated with a wide range of health outcomes, making gait health a key target for therapeutic interventions [1,2]. Gait performance can be characterized in a number of ways including, for example, speed, variability, and dynamics, each capturing distinct aspects of gait. Gait speed has received the most attention: reduced average walking velocity has been associated with lower overall quality of life, increased risk of falls and all-cause mortality, and accelerated progression of chronic diseases including diabetes, chronic heart failure, and dementia [3–5]. Increased stride time variability has also been shown to independently predict future falls in healthy ambulatory adults [6,7] and to distinguish individuals with prodromal and early stage Parkinson’s disease from healthy controls [8]. In contrast, metrics characterizing long-range gait dynamics have received less attention, even though they also show promise in uniquely informing the physiology of gait, in quantifying age-related and pathological alterations in locomotor control systems, and in augmenting objective measurement of mobility and functional status [9–13].

A growing body of research now supports the idea that when observed over longer periods of time, fluctuations in stride intervals exhibit long-range, fractal like correlations, similar to the scale free phenomena observed in long-range beat-to-beat fluctuations in heart rate [14]. This fractal like correlation may be due to the influence of multiple controls systems which influence gait. With each control system potentially operating at a different time scale some will have an immediate effect on a gait cycle while others will exert more remote, “long-range” effects. The distribution of these short and long-range influences follows a power-law which resembles fractal-like processes. Therefore metrics derived from the characteristics of fractal processes can be used to quantify the multiple physiologic processes that control gait. Using a well-validated fractal-based metric based on detrended fluctuation analysis (DFA), studies have shown that the degree of long-range correlations and fractal-like scaling in gait decreases with age in healthy adults, distinguishes patients with amytrophic lateral sclerosis, Huntington’s and later stages of Parkinson’s disease, and predicts falls in older adults with higher level gait disturbances [11,15–17]. However, to date, very few studies have evaluated the impact of either long- or short-term exercise training on gait dynamics in healthy adults. We use long-term training to mean regular and continuous Tai Chi training for at least 5 years.

Tai Chi is a multi-component mind—body exercise that is growing in popularity, especially among older adults [18–20]. Tai Chi integrates moderate aerobic conditioning along with training in balance, flexibility, and neuromuscular coordination. At the same time, Tai Chi calls in to play multiple cognitive components including heightened body awareness, focused mental attention, and imagery. Together, these multi-modal training effects may result in a range of benefits to cognition, gait health, and postural control, beyond conventional uni-modal exercise [21]. Tai Chi can improve balance and reduce fall risk in otherwise healthy and neurologically impaired older adults [22–25] and may impact multiple aspects of gait health [26–29]. Because it affects multiple physiologic systems that interact over different time scales to control gait, we postulated that a fractal-like measure, such as that which is based on DFA, may be capable of capturing changes in gait that occur in response to Tai Chi training.

A prior hybrid study design including a cross-sectional comparison between Tai Chi experts (average training > 24 years) and age- and gender-matched Tai Chi-naïve older adults reported that long-term Tai Chi training was associated with reduced stride time variability during dual task challenges [30], an outcome predictive of falls in older healthy adults [7]. Tai Chi-naïve adults in this study that were randomized to six months of Tai Chi training also exhibited modest improvements in dual task gait performance [30]. These outcomes were assessed during short bouts (90 sec) of self-selected gait speed. Using previously unreported outcomes assessed in the above hybrid study, here we analyze data derived from 10-minute bouts of self-paced continuous walking to evaluate if long- and short-term Tai Chi training impacts gait dynamics. Employing a self-similarity and scaling index derived from DFA, we specifically hypothesized that: 1) Tai Chi experts would have gait dynamics with stronger (i.e., larger) long-range and fractal-like correlations, as compared to healthy Tai Chi-naïve controls; 2) Following random assignment to six months of Tai Chi training, healthy Tai Chi-naïve adults would exhibit trends towards gait dynamics with stronger long-range and fractal-like correlations, compared with adults assigned to a wait-list control.

## Methods

### Study design

Data presented here are part of a larger investigation evaluating multiple physiological outcomes associated with long- and short-term Tai Chi training in healthy older adults. Details of the study design and population characteristics are reported elsewhere and briefly summarized below [21,30,31]. The protocol for this trial and supporting CONSORT checklist are available as supporting information; see S1 Checklist and S1 Protocol. The Institutional Review Boards of Beth Israel Deaconess Medical Center (BIDMC) and Brigham and Women’s Hospital, Boston, Massachusetts, approved this study. The randomized trial component of this study was registered at ClinicalTrials.gov (NCT01340365).

We employed a hybrid study design that included a two-arm randomized clinical trial (RCT) along with an additional observational comparison group. The Tai Chi naïve group included sixty healthy older participants, age 50–79 years, living within the Greater Boston area, and reporting no regular Tai Chi practice within the past 5 years. Participants were excluded based on the following criteria: 1) chronic medical condition including cardiovascular disease, stroke, active cancer; neurological conditions; or significant neuromuscular or musculoskeletal conditions requiring chronic use of pain medication; 2) acute medical condition requiring hospitalization within the past 6 months; 3) self-reported inability to walk continuously for 15 minutes unassisted; and 4) regular participation in physical exercise on average 4 or more times per week. Individuals who were interested underwent both an initial phone screen and an in-person screen. Those who were eligible provided written informed consent prior to outcome assessment. The participants were subsequently randomized 1:1 to receive 6 months of Tai Chi training in addition to usual healthcare, or usual healthcare alone. Study participants randomized to the usual care only group were offered a 3 month course of Tai Chi as a courtesy following the trial. Randomization was stratified by age (50–59, 60–69, 70–79 years) and utilized a permuted-blocks randomization scheme with randomly varying block sizes. Randomization was performed by the study statistician. Outcomes were assessed at baseline, 3 months and 6 months. The primary staff overseeing assessment and analysis of these gait-related outcomes was blinded to treatment assignment. Those randomized to Tai Chi were asked to attend at least 2 local Tai Chi classes per week and perform home-practice for 30 min on two additional days each week. Attendance at Tai Chi classes was recorded by the instructor and home practice was recorded using a weekly practice log.

The Tai Chi expert group consisted of twenty-seven healthy older (age 50–79 yrs) adults currently engaged in an active Tai Chi training regimen, each with at least 5 years of Tai Chi experience. There was no limitation set on the style of Tai Chi training. Eligibility and screening procedures for Tai Chi experts were identical to those for healthy adults enrolled in the RCT, with the exceptions of no limitation on exercise activity, prior Tai Chi experience, or the use of beta blockers to control diagnosed hypertension.

### Measurements

All outcome measures were assessed at the Syncope and Falls in the Elderly (SAFE) laboratory at BIDMC. The outcomes related to gait as presented here were part of a larger set of tests which lasted an average of 3.5 hours (including balance and cardiovascular function which are being reported elsewhere). Characterization of fractal dynamics between Tai Chi experts and Tai Chi naïve adults employing DFA was an a priori-defined outcome measure of primary interest.

### Assessment of gait

Gait was assessed while the subjects walked in a long unobstructed hallway. Subjects were instructed to walk at their normal preferred walking pace and make wide turns at the ends of the hallway as the 10-minute walking trial was conducted. Midway in our study, testing was changed from a longer (48m) to a shorter (23m) hallway because of access issues at the testing facility. This difference was accounted for in the statistical analyses. Footswitches (ultrathin force-sensitive resistors) were placed on the participant’s heels and toes to capture the temporal parameters of gait as they walked. Data was collected wirelessly at 1500 Hz and recorded using DTS Data Acquisition Software (Noraxon, Scottsdale, AZ). Analysis of the data was performed in Matlab (Mathworks, Natick, MA) where the initial and final foot contact times for each stride were determined. The stride interval was determined by taking the time between subsequent heel-strikes from each participant’s left foot.

We eliminated discontinuities, defined as intervals greater than 3 standard deviations (SD) away from the moving median, and stitched the waveform back together starting with the next valid point in order to focus on the “steady state” dynamics [11,15]. It has been shown that for positively correlated signals (1.5 ≥ α > 0.5), such as those observed here, stitching does not affect the scaling behavior [32]. So as to not consider pauses or stops in walking, we calculated the standard deviation based on intervals less than 2 seconds [33]. The moving median was calculated by taking the median within a centered moving window of 29 points stepped one point at a time across the time-series. In cases where a given 10-minute walking trial consisted of more than 5% discontinuities, we did not consider this trial as part of the analysis. Additionally, 5 strides at the beginning and end of a file were eliminated to account for startup and stopping issues.

### Detrended fluctuation analysis

Detrended fluctuation analysis (DFA) was used to characterize long-range gait dynamics and potential fractal properties of stride time intervals. DFA is a modified random walk analysis which uses the fact that long-range power-law correlated time series can be mapped to self-similar processes through integration [15]. As such, DFA can be used to distinguish between fluctuations due to white noise, short-range dependence and long-range correlations. Of note, DFA is relatively robust to nonstationarities since it detrends the windowed series by subtracting the locally best fit line prior to performing fluctuation analysis [34].

More precisely to estimate the DFA alpha coefficient for a time-series ${\left\{x\left(i\right)\right\}}_{i=1}^{N}$ of length *N* we perform the following steps [14,35]:

1. Integrate the series’ deviations from its mean, $\bar{x}$, which yields $y_{i}=\sum_{j=1}^{i}\left|x\left(j\right)-\bar{x}\right|$ for *i* = 1,…, *N*.
2. For a given box size *n*, divide ${\left\{y\left(i\right)\right\}}_{i=1}^{N}$ into ⌊*N*/*n*⌋ non-overlapping boxes of length, *n*, where ⌊.⌋ represents the greatest integer function.
3. Fit a least squares line to the data within each box. The resulting sequence of fitted lines constitutes the trend series ${\left\{y_{n}\left(i\right)\right\}}_{i=1}^{N_{n}}$ where *N*_*n*_ = *n*⌊*N*/*n*⌋ is the total number of data points falling within the boxes.
4. Calculate the average fluctuation, *F*(*n*), of the integrated series *y* around the trend series *y*_*n*_. More explicitly, $F\left(n\right)=\;\sqrt{\frac{1}{N_{n}}\sum_{i=1}^{N_{n}}{\left|y\left(i\right)-\;y_{n}\right|}^{2}}$.
5. Repeat steps 2–4 above for different box sizes *n*_1,_…,*n*_*m*_, to get a range of fluctuations *F*(*n*_1_),…,*F*(*n*_*m*_).
6. Plot log *F*(*n*_*i*_) versus log *n*_*i*_, for *i* = 1,…,*m*. For an obvious linear relationship between them, the least squares fitted slope provides the estimated scaling coefficient α.

The DFA analyses of gait dynamics presented here generally followed approaches employed in our prior studies [11,33]. In the present analysis, the window size (n) varied from 16 to 58. Our choice of the window size range was based on work by Damouras et al. who analyzed bouts of walking of a similar duration (15 min) [35]. In an effort to make an accurate estimate of alpha, their method eliminated small and large window sizes that exerted too much influence (based on a statistical test of standardized difference). They recommended using a range of 16 –N/9, where N is the number of stride intervals. Our median data length was 527 stride intervals. To ensure analysis of the same time-scales across subjects, we fixed our maximum box size at 58 based on the median divided by 9. The integrated time series is said to be self-similar if the fluctuations at different observation windows [F(n)] scale as a power law with the window size [15]. In our analysis, n corresponds to the number of strides in the window.

Generally, F(n) will increase with window size n. If plotting log F(n) against log (n) across the various window sizes produces a linear relationship, the slope of the best fit line is taken as the self-similarity index or scaling index (α). For a completely uncorrelated process (such as white noise), α = 0.5. Processes with consistent long-range correlations produce 0.5< α <1. An α = 1 is observed for 1/f noise which lies on the boundary between stationary (α<1) and nonstationary (α>1) processes [34].

Average gait speed (m/s) was calculated based on the distance walked during the same 10-minute walking bout used to analyze gait dynamics.

### Baseline characteristics

Sociodemographic variables including age, gender, and race were collected at baseline. Baseline physical activity level was assessed using the physical activity status scale (PASS). The scale quantifies physical activity duration by a combination of the minutes of exercise per week and the intensity of this exercise (i.e., heavy, modest, or none) [36]. Baseline global cognitive function was assessed with the Mini Mental State Exam [37]. Because of the well-established impact of executive function on various aspects of gait [7,38] we also evaluated baseline executive function using two widely used measures, Category Fluency and the Trail Making Test [39,40]. Results of these two outcomes were combined to create a composite executive function (EF) Z-score using methods described elsewhere [41].

### Statistical analysis

Measures of DFA in Tai Chi experts and Tai Chi naïve subjects were compared using a linear modeling approach. Model 1 presents the unadjusted results and Model 2 is adjusted just for hallway length. Because of the significant impact of hallway length, this confounder was included in all subsequent models. Because of the observational, non-randomized nature of this comparison, age and gender were a priori defined potential confounders, thus Model 3 includes age and gender. Model 4 includes body-mass-index and physical activity, which differed at baseline, in addition to Model 3 confounders. Finally, Model 5 also included an executive function Z-Score, in addition to Model 4 confounders. Effect sizes were calculated by taking the ratio of the modeled alpha estimate difference between the Tai Chi experts and Tai Chi naïve groups relative to the standard deviation of alpha in the usual care group.

Differences between groups randomized to Tai Chi versus Usual Care were assessed using a linear mixed model that included random slopes and a shared baseline. These analyses were conducted according to an intention-to-treat paradigm. All models included fixed effect of time and time × treatment and random participant-specific intercepts and slopes with unstructured covariance. Model 1 included only these effects. Model 2 also included fixed effect of hallway length and time × hallway length. In addition to the effects included in Model 2, Model 3 also included age, time × age, gender, and time × gender. The shared baseline assumption, enforced by omitting a treatment main-effect term, properly reflects the true state of the population sampled prior to randomization and has the advantage of adjusting for any chance differences at baseline [42]. We recorded gait speed and evaluated it as a potential confounder in our analysis, but differences were very small, and its inclusion did not significantly impact our statistical models. Thus speed was not included in our final cross-sectional or longitudinal models. Effect sizes were calculated by taking the ratio of the modeled alpha slope difference between those randomized to Tai Chi versus Usual Care relative to the standard deviation of the change in alpha from baseline to 6 months in the usual care group subjects who completed their 6 month visit. Associations between changes in DFA alpha vs. dosage of Tai Chi training were estimated using linear regression.

Treatment-group differences and adjusted means were estimated along with their 95% confidence intervals. All inferential tests were two-tailed at alpha = 0.05. We choose to report comparison-wise p-values without adjustment for multiple comparisons to avoid inflating type II errors, recognizing that the nominal p-values underestimate the overall experiment-wise type I error rate.

To assist in the interpretation of effect sizes, it is generally taken that an effect size of 0.5 is considered medium while an effect size of 0.2 is considered small and an effect size of 0.8 is considered large [43].

Our results are intended as hypothesis generating, not definitive tests of efficacy of Tai Chi on any specific measure of gait. All analyses were performed in SAS (version 9.3, SAS Institute, Cary, NC).

Regarding sample size considerations, for cross-sectional comparisons, we estimated that a sample size of 27 Tai Chi Expert and 60 Tai Chi naïve subjects would provide power to detect an effect size of 0.63. For the randomized trial, we estimated that the sample of 30 individuals per group would ensure at least 80% power for a treatment effect equal to an effect size of 0.73.

## Results

### Participant characteristics

Tai Chi experts (n = 27) had an average of 24.5±11.8 years of Tai Chi training (median 20 yrs, range 10–50 yrs). The number of experts reporting Yang and Wu style as their primary training system, n = 12 and n = 15 respectively, were nearly equal. Tai Chi experts and naïve subjects were generally well matched with respect to average age and global cognitive status. However Tai Chi experts had lower BMI (body mass index) and higher levels of physical activity, and included a slightly higher proportion of men and Asians. Tai Chi naïve participants subsequently randomized to Tai Chi plus usual care versus usual care alone were comparable at baseline (Table 1).

**Table 1:** Group characteristics.

	Observational Groups		Randomized Groups	
	Tai Chi experts	Tai Chi naïve	Usual Care	Tai Chi
	(n = 27)	(n = 60)	(n = 29)	(n = 31)
**Age**^a^ (yrs)	62.78 ± 7.57	64.18 ± 7.68	64.45 ± 7.42	63.94 ± 8.02
**Gender** n (%)				
Male	13 (48.1%)	20 (33.3%)	11 (37.9%)	9 (29%)
Female	14 (51.9%)	40 (66.7%)	18 (62.1%)	22 (71%)
**Race** n (%)				
White	22 (81.5%)	55 (91.7%)	26 (89.7%)	29 (93.5%)
African American	1 (3.7%)	3 (5%)	3 (10.3%)	0 (0%)
Asian	4 (14.8%)	2 (3.3%)	0 (0%)	2 (6.5%)
**Ethnicity** n (%)				
Non-Hispanic/Non-Latino	26 (96.3%)	59 (98.3%)	29 (100%)	30 (96.8%)
Hispanic/Latino	1 (3.7%)	1 (1.7%)	0 (0%)	1 (3.2%)
**Education (yrs)** ^a^	18.44 ± 3.34	16.7 ± 3.25	16.19 ± 3.03	17.13 ± 3.41
**MMSE (out of 30)** ^a^	29.07 ± 1.11	29.12 ± 1.01	29.21 ± 0.82	29.03 ± 1.17
**BMI (kg/m**^**2**^**)** ^a^	23.54 ± 2.35	26.46 ± 5.46	26.54 ± 5.83	26.38 ± 5.19
**Physical Activity Level**^a^^,^^b^	6.0 ± 2.0	4.4 ± 2.2	4.0 ± 2.0	4.0 ± 2.0
**Executive Function Z-score**^c^	0.28 ± 1.2	-0.12 ± 1.3	-	-
**Exposure to Tai-Chi** hours^a^ (% of subjects)				
Compliant subjects^d^	-	-	-	89 ± 25 (48%)
Non-compliant subjects^d^	-	-	-	34 ± 25 (52%)

^a^ values provided are mean ± standard deviation

^b^ Physical Activity Level descriptions:

4 = Run about 1 mile per week OR walk about 1.3 miles per week OR spend about 30 minutes per week in comparable physical activity.

5 = Run about 1 to 5 miles per week OR walk 1.3 to 6 miles per week OR spend 30 to 60 minutes per week in comparable physical activity.

6 = Run about 6 to 10 miles per week OR walk 7 to 13 miles per week OR spend 1 to 3 hours per week in comparable physical activity.

^C^ Executive Function Z-score was calculated from the ratio of the standardized Trail Making and COWAT tests. More specifically the ratio of Trail Making B to Trail Making A and Category Fluency were used to generate an Executive Function Z-score.

^d^ Participants that attended a minimum of 70% of all classes (two 1 hour classes per week) and completed 70% or more of prescribed home practice (two 30 minute sessions per week) between each study visit were considered compliant.

### Recruitment and protocol adherence

**Fig 1:**
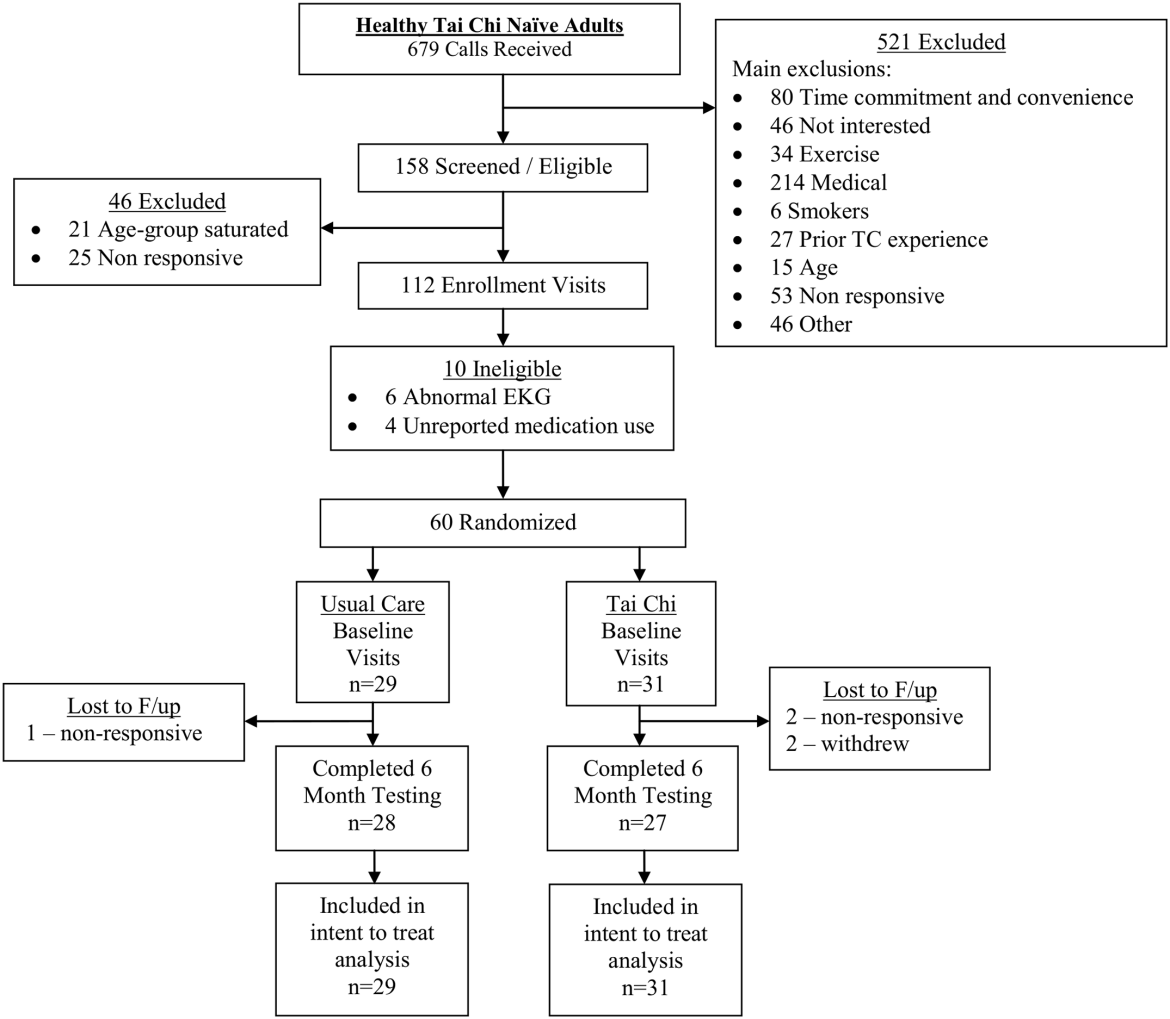
Participant flow through the randomized trial sub-study.

Participant flow through the randomized trial component of the study is summarized in Fig 1. Recruitment spanned from March 2011 to March 2013. All follow up procedures were completed by September 2013. Sixty healthy adults were successfully screened and enrolled, and 97% (28/29) and 87% (27/31) of individuals in the usual care and Tai Chi group completed the primary 6-month follow-up assessment, respectively.

A total of 4 non-serious adverse events were reported throughout study. All events were reported by participants randomized to the Tai Chi group. Only 2 of the 4 events were determined to be related to the Tai Chi intervention (both minor musculoskeletal injuries (one wrist, one ankle)).

### Cross-sectional comparisons

#### Detrended fluctuation analysis

**Fig 2:**
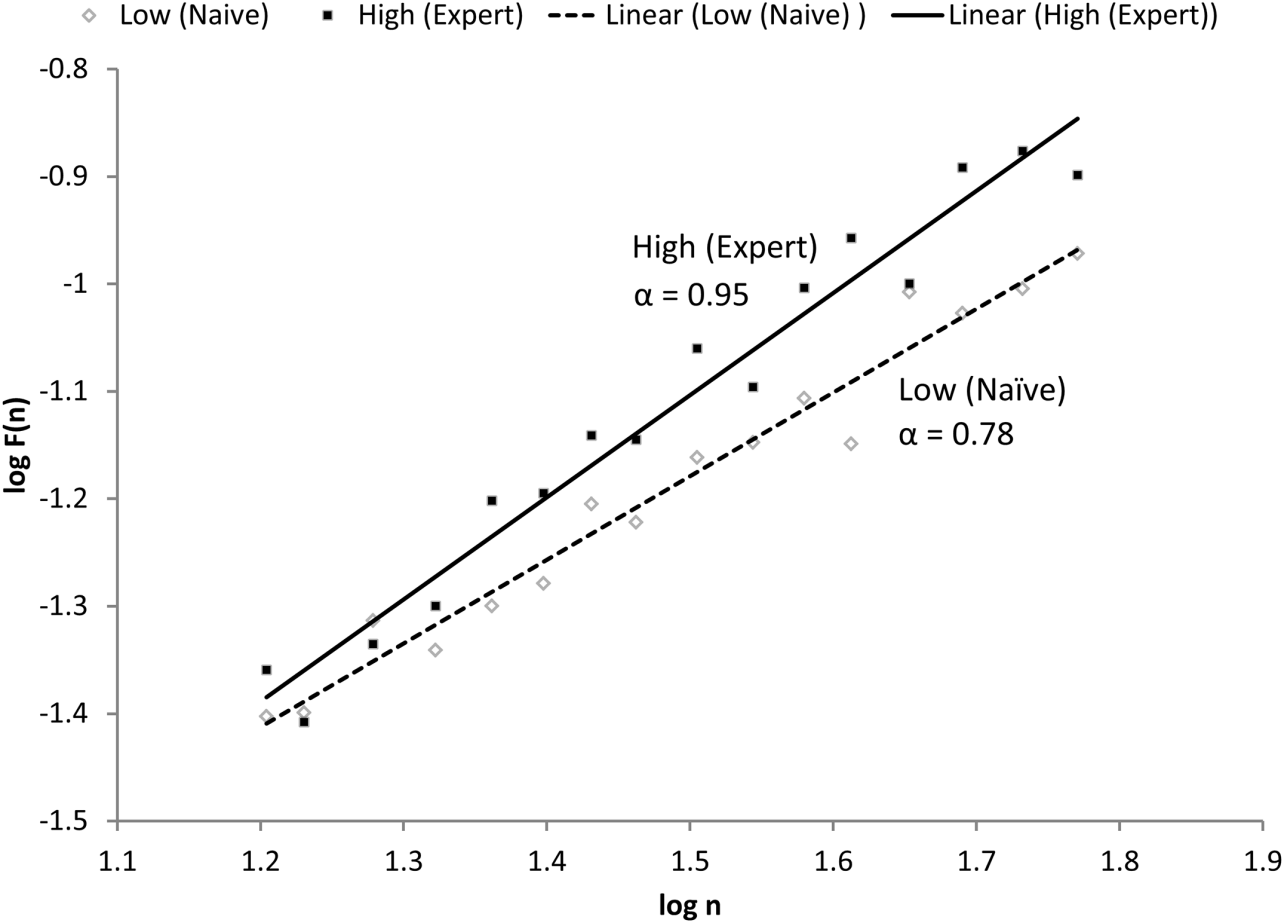
Example plots of a high (expert with α = 0.95) and low (naïve with α = 0.78) DFA alpha (α) result. The slope of the linear best-fit line on the log F(n) versus log n plot represents alpha.

Including both Tai Chi expert and naïve groups and all outcomes times, filtered 10 minute gait time-series resulted in a mean of 527 strides with a standard deviation of 46 where the minimum number of points across time series was 425. Fig 2 illustrates example best-fit linear plots of log F(n) versus log (n) plots from which alpha coefficients are derived; examples contrast a Tai Chi expert (upper quartile) and a Tai Chi naïve (lower quartile) subject.

Across all statistical models, mean estimated alpha coefficients ranged from 0.841 to 0.925, indicating long-range correlations and fractal scaling in stride time variability (Table 2). Tai Chi experts displayed a higher degree of self-similarity or fractal-like dynamics than Tai Chi naïve in all models. Model 3 which included adjustments for age, gender and hallway length indicated a statistically significant difference in alpha coefficients between Tai Chi experts and Tai Chi naïves (0.907 vs. 0.849; p = 0.027). Model 4 which included the additional potential confounders of BMI and physical activity indicated larger between-group differences in alpha (0.919 vs. 0.845; p = 0.007). Finally, the further addition of executive cognitive function as a potential confounder in Model 5 led to an even greater between group difference in alpha and the largest effect size in the models tested (0.925 vs. 0.841; effect size = 0.72).

**Table 2:** Estimated mean values and 95% confidence intervals for alpha coefficients derived from detrended fluctuation analyses for Tai Chi expert and Tai Chi naïve cross-sectional comparison groups.

Statistical Model	Groups				Goodness of Fit					
	Tai Chi naïve (n = 60)	Tai Chi experts (n = 27)								
	Mean (95% CI)	Mean (95% CI)	p-value	Effect size	Deviance	AIC	BIC	Models Compared	Δ DF	Chi^2^ p-value
1	0.868 (0.835, 0.901)	0.901 (0.854, 0.948)	0.256	0.28	-113	-107	-100			
2	0.860 (0.829, 0.891)	0.913 (0.869, 0.957)	0.056	0.45	-125	-117	-107	2:1	2	0.003
3	0.849 (0.819, 0.879)	0.907 (0.865, 0.950)	0.027	0.50	-135	-123	-108	3:2	3	0.016
4	0.845 (0.815, 0.874)	0.919 (0.876, 0.962)	0.007	0.63	-139	-123	-103	4:3	2	0.165
5	0.841 (0.811, 0.871)	0.925 (0.882, 0.969)	0.004	0.72	-140	-122	-100	5:4	1	0.237

Results for five linear models, each including increasing numbers of potential confounders are shown. Model 1 is unadjusted. Model 2 adjusts for hall length. Model 3 adjusts for age, gender and hall length. Model 4 adjusts for BMI and levels of physical activity, in addition to model 3 confounders. Model 5 adjusts for executive function, in addition to Model 4 confounders. Goodness of fit statistics, residual deviance (-2 log likelihood), Akaike information criterion (AIC), and Bayesian information criterion (BIC), are provided for each model. More negative values indicate better fit. The difference in degrees of freedom (ΔDF) and Chi^2^ p-values from likelihood ratio tests comparing sequential models are given. Effect sizes were calculated relative to the standard deviation of the usual care group.

#### Gait speed and executive cognitive function

Gait speed (in m/sec) averaged over the 10 minutes of walking was nearly identical in the two groups (expert 1.100: CIs 1.046, 1.155, naïve 1.103: CIs 1.065, 1.142)(p = 0.929) when adjusting for age, gender, BMI, physical activity and hallway length. Executive function, as assessed using a Z-score combining the Category Fluency and Trail Making B/A outcomes, indicated a statistically non-significant trend of Tai Chi experts exhibiting modestly better cognitive ability (p = 0.185).

### Longitudinal comparisons

#### Detrended fluctuation analysis

Across all fitted models for both randomly assigned groups, estimates of changes in the alpha coefficient over 6 months were modest and ranged from 0.002 to 0.052. Average group changes of the slope of the alpha coefficient across 6 months were consistently greater (i.e., suggesting higher fractal scaling) in those randomized to Tai Chi vs. usual care, however, between group differences were small and not statistically significant (Table 3). Additionally the very small effect sizes suggest a lack of clinical significance. Model 3 which adjusted for baseline age, gender and hallway length indicated a group difference in alpha at 6 months of only 0.011 (p = 0.348). Change in alpha in the Tai Chi group at 6 months estimated by model 3 was 0.002. For reference, this is more than an order of magnitude smaller than the between group difference observed in the cross-sectional component of this study (0.058).

**Table 3:** Estimated mean slope values (estimated change per visit) and 95% confidence intervals for changes in DFA alpha coefficients for participants randomly assigned to six months of Tai Chi training plus usual care or to usual care alone.

Statistical Model	Groups				Goodness of Fit					
	Tai Chi (n = 31)	Usual Care(n = 29)								
	Mean (95% CI)	Mean (95% CI)	p-value	Effect size	Deviance	AIC	BIC	Models Compared	Δ DF	Chi^2^ p-value
1	0.017 (-0.012, 0.046)	0.007 (-0.020, 0.035)	0.423	0.050	-212	-198	-184			
2	0.028 (0.001, 0.056)	0.017 (-0.009, 0.044)	0.325	0.059	-226	-208	-189	2:1	4	0.009
3	0.009 (-0.022, 0.040)	-0.002 (-0.032, 0.028)	0.348	0.056	-237	-211	-184	3:2	4	0.025

Model 1 is unadjusted. Model 2 adjusts for hall length. Model 3 adjusts for age, gender and hall length. Goodness of fit statistics, residual deviance (-2 log likelihood), Akaike information criterion (AIC), and Bayesian information criterion (BIC), are provided for each model. More negative values indicate better fit. The difference in degrees of freedom (ΔDF) and Chi^2^ p-values from likelihood ratio tests comparing sequential models are given. Effect sizes were calculated relative to the standard deviation of change scores from baseline to six months for the usual care group subjects who completed their six month follow-up.

A post-hoc analysis limited to participants randomized to 6 months of Tai Chi training showed a positive correlation between the change in the alpha coefficient versus total hours of Tai Chi exposure (combined class and home training), but this trend was not statistically significant (p = 0.201).

#### Gait speed and executive function

Magnitudes of changes in gait speed and z-scores for executive cognitive function at 6 months were small and did not differ significantly between groups (Table 4).

**Table 4:** Estimated mean slope values (estimated change per visit) and 95% confidence intervals for changes in gait speed and executive function z-score for participants randomly assigned to six months of Tai Chi training plus usual care or to usual care alone.

Variable	Groups		p-value
	Tai Chi (n = 31)	Usual Care(n = 29)	
	Mean (95% CI)	Mean (95% CI)	
Gait speed (m/s)	-0.001 (-0.026, 0.024)	0.011 (-0.013, 0.036)	0.434
Executive function Z	0.224 (-0.009, 0.457)	0.212 (-0.018, 0.442)	0.918

Results are from unadjusted models.

## Discussion

One increasingly accepted interpretation of long-range fractal-like correlations in physiological systems is that they reflect the degree of non-linear complexity, which in turn, represents adaptability and the capacity to respond to unpredictable stimuli and stresses [44,45]. With aging and disease, this aspect of complexity is altered in a number of physiological signals including heart rate, blood pressure, and breathing, predicts important outcomes, and is sensitive to certain therapeutic interventions [31,44–46]. In this sense, long-range correlations reflect (self-)organizing mechanisms of highly complex processes that generate fluctuations across a wide range of time scales, where the absence of a characteristic scale inhibits the emergence of highly periodic behaviors, which would greatly narrow functional responsiveness. In this context, the association of lower DFA scaling indices and risk of falling among individuals with both Parkinson’s disease and higher-level gait disorders may reflect a reduced resilience or capacity in these populations to adapt to fall-related perturbations [11]. More generally, lower degrees of scaling in gait, may reflect, in part, less flexible neurobiological underpinnings.

While the loss of fractal-like stride dynamics during gait has been previously associated with aging and disease [11,15–17], little research has evaluated the plasticity of this gait property or the potential for exercise interventions to positively impact long-range stride time dynamics. The cross-sectional component of this hybrid study is the first to report an association between long-term Tai Chi mind-body exercise training and greater fractal-like correlations in stride intervals, as reflected in higher self-similarity and scaling indices (α) derived from DFA. These novel results suggest that Tai Chi is associated with healthy gait dynamics as indicated by a higher degree of fractal scaling in Tai Chi experts compared to the Tai Chi naive. Nonetheless, cause and effect in the observational component of this study cannot be definitively ascribed. In comparison, the short-term effects of six months of Tai Chi training on fractal-like dynamics observed in the randomized component of this study, while trending in the direction of greater self-similarity, were small and not statistically significant. Additionally, among individuals randomly assigned to Tai Chi, there was a non-statistically significant positive association between levels of Tai Chi training exposure (i.e., class and home practice time) and greater fractal-like dynamics. Collectively, these results suggest that fractal-like measures of gait may be informative in characterizing longer-term training impacts of Tai Chi, but further research is required to evaluate their utility in assessing the impact of short-term Tai Chi training in both healthy and health-impaired populations.

A noteworthy finding in this present study is that, in contrast to group differences in DFA scaling coefficients, we observed no between-group differences in preferred gait speed averaged over the 10 minutes of walking in either the cross-sectional or longitudinal comparisons. This finding parallels results reported in a previous study using the same experimental design. In that study [30], quiet walking during 90 sec bouts also did not differ between Tai Chi experts vs. Tai Chi naïve controls (and walking speed was nearly identical suggesting fatigue was not a factor contributing to observed speeds in this study), but groups did differ in measures of stride time variability during dual task challenges (Tai Chi experts had lower and “better” variability). Other studies in older adults have also reported no impact of Tai Chi on walking speed, even when other measures of function or fall risk were improved [47–49], although multiple studies have also reported positive effects of Tai Chi on gait speed [50–54].

The participants in this study were very healthy and fall histories were not recorded (and likely were very low). Still, we speculate that the higher DFA scaling index in the Tai Chi expert group is associated with greater locomotor control and a lower risk of future falls, helping to explain the positive impact of Tai Chi on postural control and fall prevention reported in multiple clinical trials [22,54,55]. Collectively, these findings highlight the need for research exploring the utility of novel measures of gait for characterizing health and predicting future events (e.g., falls) should not be driven by the goal of identifying a single metric. Rather, any new metric should be viewed as one element in a matrix of complementary outcomes of gait, each evaluating and informing different aspects of the neurophysiological control of gait and overall health [9].

The physiological basis for long-range correlations of gait dynamics are not fully understood, but multiple theories and a growing body of empirical evidence supports a key role for central nervous system mechanisms. Gait relies on components from numerous feedback and feedforward loops across multiple time scales for motor control and timing including those from the neural-muscular periphery, the intraspinal nervous system, basal ganglia and central networks. A breakdown in one of these systems such as the basal ganglia can reduce the healthy (1/f) fractal dynamics and lead to compromised locomotor function [56]. One theory emphasizes the role of supra-spinal control and posits that the simultaneous functioning of multiple neural networks contribute to this fractal-like scaling. Self-regulation and corrections across multiple time scales can lead to this complex output. These theories are supported by models and studies as well as by studies showing scaling is unchanged in peripheral disease and improved by methylphenidrate, which primarily impacts the CNS (central nervous system) [11,57]. Further, some evidence suggests that a difficult dual task, i.e. significant cognitive loading, also diminishes fractal-like gait scaling [11,57,58]. These findings are all consistent with idea that fractal-like scaling has higher-level origins. As noted above, as a multimodal mind-body exercise, Tai Chi likely imparts its observed therapeutic effects through impact on both central and peripheral processes [21,31]. Future translational research utilizing CNS imaging tools and comparing the impact of multimodal mind-body exercises like Tai Chi to relatively simple unimodal exercises (e.g., lower extremity strength training) might shed insight into the mechanisms through which Tai Chi impacts gait health, including long-range gait dynamics. A better understanding of CNS plasticity in response to exercise interventions will greatly enhance the effectiveness of this exercise program alone, or in combination with other pharmacological and non-pharmacological approaches, in rehabilitation and prevention.

The observed effect size in the longitudinal comparison of our study was quite small. Based on scaling up from the sample size of 60 in this study and the observed t-statistic in model 3 of 0.95, a follow-up trial with the same design and equivalent effects of treatment and covariates, equivalent variance terms, and equivalent rates of treatment adherence and loss to follow-up would require a sample size of 522 for 80% power to declare significance with a two-tailed p < 0.05. Given the small effect size observed in the exploratory longitudinal study of very healthy adults, we believe that a larger study in this population is not warranted. Rather, a future study evaluating the impact of Tai Chi training on gait dynamics would be more logical in a population of older adults with clear gait abnormalities (e.g., Parkinson’s disease) or with higher risks of falling. Such a study should also include longer training periods (e.g., 12 months) and multiple assessment points to better evaluate the possible effect of exposure duration on outcomes.

Future clinical trials and large-scale longitudinal observational studies are needed to better evaluate whether Tai Chi training enhances fractal-like gait dynamics, and whether such changes in gait dynamics are associated with improved locomotor control and reduced falls.

Our study has important limitations. First, regarding our cross-sectional comparisons, both the Tai Chi expert and Tai Chi naïve populations were small and statistical comparisons could have resulted in type II errors. Second, the between group difference we observed in α values appear to be modest. However, because α is estimated on a log-log scale, the magnitude of observed differences in cross-sectional comparisons are actually relatively large, and within the same range observed when DFA is used in studies of heart rate and other physiological dynamics that are associated with and predict significant outcomes [45,59]. Third, as with any cross-sectional study, comparisons between groups may be confounded by differences between groups other than Tai Chi exposure. Nevertheless, the observed effect of Tai Chi training was apparent even after accounting for several confounders, including executive function. Limitations for the randomized trial component of this study include small sample sizes and relatively short-term exposures. The short-term nature of Tai Chi training is especially relevant given that our population was already very healthy and physically active.

## Conclusions

Findings from cross-sectional, and to a lesser extent, randomized group comparisons, suggest that Tai Chi training is positively associated with higher fractal-like gait dynamics, an established marker of gait health. Larger and longer-duration randomized trials are needed, in both healthy and health challenged adult populations, to evaluate the potential of Tai Chi to restore age-related declines in indices of fractal-like gait dynamics.

## Supporting information

S1 Checklist CONSORT checklist. (DOC)

S1 Protocol Trial protocol. (DOCX)

## Abbreviations

BIDMC: Beth Israel Deaconess Medical Center
BMI: Body Mass Index
CNS: central nervous system
DFA: Detrended Fluctuation Analysis
PASS: physical activity status scale
SAFE: Syncope and Falls in the Elderly
TC: Tai Chi
